# Effect of community-based education on undergraduate nursing students’ skills: a systematic review

**DOI:** 10.1186/s12912-021-00755-4

**Published:** 2021-11-18

**Authors:** Arezoo Zeydani, Foroozan Atashzadeh-Shoorideh, Fatemeh Abdi, Meimanat Hosseini, Sima Zohari-Anboohi, Victoria Skerrett

**Affiliations:** 1 Student Research Committee, School of Nursing and Midwifery, Shahid Beheshti University of Medical Sciences, Tehran, Iran; 2 Department of Psychiatric Nursing and Management, School of Nursing and Midwifery, Shahid Labbafinezhad Hospital, Shahid Beheshti University of Medical Sciences, Tehran, Iran; 3 School of Nursing and Midwifery, Alborz University of Medical Sciences, Karaj, Iran; 4 Department of Community Health Nursing, School of Nursing and Midwifery, Shahid Beheshti University of Medical Sciences, Tehran, Iran; 5 Department of Medical Surgical-Nursing, School of Nursing and Midwifery, Shahid Beheshti University of Medical Sciences, Tehran, Iran; 6 School of Nursing and Midwifery, Birmingham City University, Birmingham, UK

**Keywords:** Education, Community-based, Skill, Student, Nursing

## Abstract

**Background:**

Community-based education, as an effective approach to strengthen nurses’ skills in response to society’s problems and needs has increased in nursing education programs. The aim of this study was to review the effect of community-based education on nursing students’ skills.

**Methods:**

For this systematic review, ProQuest, EMBASE, Scopus, PubMed/ MEDLINE, Cochran Library, Web of Science, CINAHL and Google Scholar were searched up to February 2021. The methodological quality of the studies was assessed using the Mixed Methods Appraisal Tool (MMAT). Seventeen studies were included in this systematic review. Inclusion criteria included articles published in English and were original articles.

**Results:**

In all studies, undergraduate nursing students’ skills were improved by participation in a community-based education program. Community-based education enhances professional skills, communication skills, self-confidence, knowledge and awareness, and critical thinking skills and teamwork skills in undergraduate nursing students.

**Conclusions:**

Community-based education should be used as an effective and practical method of training capable nurses to meet the changing needs of society, to improve nurses ‘skills and empower them to address problems in society.

## Background

The main mission of nursing education is to train competent and confident nurses with the knowledge, attitude and skills necessary to maintain and promote community health [1, 2]. The main purpose of nursing education is to develop critical thinking, creative thinking, reflective learning, professional skills, time management, self-esteem and effective communication [3]. However, many nursing graduates do not have advanced skills in communication, creativity, critical and analytical thinking, problem solving, and decision making. Therefore, nurses should be empowered to meet the needs of society [1, 4].

It has been proven that traditional teaching methods are not fully effective in improving the cognitive skills and abilities of nursing students [5], as this method does not address the needs, changes and problems of the society. Challenges such as increase in emerging diseases, increase in chronic diseases, aging population and advances in technology require nurses who not only have advanced knowledge but also have higher thinking skills such as critical thinking, problem solving and decision making [6]. Nurses have the potential to be a powerful resource for creating a healthy population and promoting economic and social development [7], and community nurse participation is central to this public health impact [8].

Several countries follow a community-based education program to cover the role of nurses in public health. Community-based education (CBE) has several definitions, but the core definition refers to learning that takes place in a setting outside the higher education institution. CBE refers to education in which trainees learn and acquire professional competencies in a community setting [9]. Internationally, changes to education have taken place. For example, the South African government called for a shift in health care education from a traditional content-based approach to a community-based approach so that students and educators could experience it [10]. UK health policy has emphasized community-based care in recent years because it has been shown to increase nurses’ competence and confidence [11]. In the United States, community-based education has also had a positive impact on students by improving their skills and increasing their understanding and responsibility [12]. In Iran, health care systems are changing to address the needs of stakeholders, cost-effective care requirements, quality improvement, and community health improvement [13].

The role and scope of nursing practice have evolved in response to the changing needs of individuals, communities and health services. The increasing aging of the population, the number of people with chronic conditions, and the emergence of new diseases have necessitated changes in service provision [14]. The role of health professionals is changing worldwide with the goal of “health for all” through “primary health care“[15].

Community-based education seems to be a promising approach to improve the relationship between education and the needs of the population. This education can increase students’ skills, as it is based on the philosophy of “primary health care”, Community-based education utilizes the community as a learning environment in which not only students, but also nursing educators, community members, and representatives from other sectors actively participate in the learning experience[15]. Community-based nursing education programs are necessary to prevent, maintain, and promote community health. At the same time, it promotes personal, social, psychological growth, and increases the skills of innovation, communication and critical thinking development in students as they see the context in which health and illness occur. It provides opportunities for nursing students to learn more about the socioeconomic, political, and cultural aspects of health and illness in society [16].

Given the importance of the role of nurses in meeting the needs of society and maintaining and promoting community health, it is important to train capable nurses with the necessary skills for society. Community-based education programs in nursing in Iran have received much attention recently [14], Several studies have been conducted on the effects of community-based education programs on nurses’ skills, but to date there has been no systematic review that comprehensively and separately examines the effects of community-based education on the undergraduate nursing students’ skills. This study comprises a systematic review of research on community-based education for nurses, the findings of which can be used to develop teaching programs. The aim of this review study was therefore to provide an accurate overview of the effect of community-based education on the undergraduate nursing students’ skills.

## Methods

This systematic review was conducted in accordance with the Preferred Reporting Items for Systematic Reviews and Meta-Analyzes (PRISMA) guidelines for the design and conduct of systematic reviews. The following steps were taken: a systematic literature search, organization of documents for review, data extraction and quality assessment of each study, synthesizing data, and writing of the report.

### Search strategy

The keywords were “community-based”, “education”, “skills”, “nursing”, and “student”, which were searched individually and in combination with AND/OR (Table 1). The systematic literature search was performed in databases such as Scopus, PubMed / MEDLINE, ProQuest, Web of Sciences, CINAHL, Google Scholar, Cochran Library and EMBASE up to February 2021. Inclusion criteria included articles published in English and were original articles. The search terms were obtained from published studies, primary studies and via PubMed MeSH. According to the PICO framework for formulating clinical questions, the queries include four aspects: Patient-Problem (P), Intervention (I), Comparison (C) and Outcome (O). In this regard, Population, Intervention, Comparators, Outcomes, Study Design (PICOS) criteria were used for this study: population (undergraduate nursing students), intervention (community-based education) and outcome (impact on skills).

**Table 1:** Search strategy

ID	Search term
#1	“Community-based” [tiab], OR “ Community-oriented” [tiab],
#2	“Education” [tiab], OR “Learning” [tiab], OR “Training” [tiab],
#3	“Student” [tiab], OR “Students” [tiab],OR “Undergraduate Students” [tiab], OR “Bachelor Students” [tiab],
#4	“Nursing” [tiab], OR “Take Care” [tiab],
#5	“Skill” [tiab] OR “Competency” [tiab]
#6	(#1 AND #2); (#1 AND #3); (#1 AND #4); (#1 AND #5); (#1 AND #2 AND #3 AND #4 AND #5)

### Study selection

Identified reports were downloaded to a library database. First, the titles and abstracts of the articles and the studies under consideration were reviewed for the match with inclusion criteria. Two authors independently reviewed the full text of the articles and discussed discrepancies until agreement was reached. Study details were extracted from articles and charted in a table which was used to make a decision about study inclusion.

### Inclusion criteria

Inclusion criteria included a focus on nursing students’ skills, community-based education, and publication of articles in English. Due to the limited number of studies involving community-based education interventions, studies using quantitative, qualitative, and combined methods were considered.

### Exclusion criteria

Articles relating to hospital education and articles presented at conferences, congresses, or in the form of books and letters to the editor were excluded.

### Data extraction

At this stage, 90 potential studies were listed. After 53 duplicates were removed, another researcher reviewed the remaining articles simultaneously and separately. Eleven studies were excluded because the title and content did not match the topic. In addition, nine studies were excluded from the study because access to the full text of the articles was not available. Seventeen articles were included in the analysis.

### Quality assessment process

The methodological quality of the included studies assessed independently by two authors using the Mixed Methods Appraisal Tool (MMAT) [17]. The MMAT was designed to assess various empirical studies in five categories, including qualitative studies, randomized controlled trials, non-randomized studies, quantitative-descriptive studies, and mixed methods studies. This instrument consists of 5 items for each category, each of which could be marked as yes, no, or not known. The scoring system provides that the “yes” answer is scored as 1 and all other answers are scored as 0. A higher score indicates higher quality. When evaluating the final scores in terms of quality, scores above half (more than 50 %) were considered high quality [18] (Table 2). Finally, the data were analyzed by extracting the textual content of the articles in the context of the study question.

**Table 2:** Appraising of the selected studies based on Mixed Method Appraisal Tool (MMAT); Version 18

**Selected** **Studies**	**Appraisal Quality**	**Quantitative non-randomized Criteria**				
		Are the participants representative of the target population?	Are measurements appropriate regarding both the outcome and intervention (or exposure)?	Are there complete outcome data?	Are the confounders accounted for in the design and analysis?	During the study period, is the intervention administered (or exposure occurred) as intended?
Nowak. 2015 [19]	H	Y	Y	Y	N	Y
Lubbers et al. 2016 [20]	H	Y	Y	Y	N	Y
Lubbers et al. 2017 [21]	H	Y	Y	Y	N	Y
Higgins.2020 [22]	H	Y	Y	Y	N	Y
Cheng et al. 2020 [23]	H	Y	Y	Y	N	Y
		**Quantitative descriptive Criteria**				
		Is the sampling strategy relevant to address the research question?	Is the sample representative of the target population?	Are the measurements appropriate?	Is the risk of nonresponse bias low?	Is the statistical analysis appropriate to answer the research question?
De Villiers et al. 2004 [24]	H	Y	Y	Y	Y	Y
Ibrahim.2010 [12]	H	Y	C	Y	Y	C
Wee et al. 2010 [25]	H	C	C	Y	Y	Y
		**Mixed methods Criteria**				
		Is there an adequate rationale for using a mixed-methods design to address the research question?	Are the different components of the study effectively integrated to answer the research question?	Are the outputs of the integration of qualitative and quantitative components adequately interpreted?	Are divergences and inconsistencies between quantitative and qualitative results adequately addressed?	Do the different components of the study adhere to the quality criteria of each tradition of the methods involved?
Street et al. 2007[26]	H	Y	Y	Y	Y	Y
Mwanika et al. 2011 [27]	H	N	Y	Y	Y	Y
Parry et al. 2018 [28]	H	Y	Y	Y	Y	Y
Lestari et al. 2020 [29]	H	Y	Y	Y	Y	Y
		**Qualitative Criteria**				
		Is the qualitative approach appropriate to answer the question?	Are the qualitative data collection methods adequate to address the research question?	Are the findings adequately derived from the data?	Is the interpretation of results sufficiently substantiated by data?	Is there coherence between qualitative data sources, collection, analysis and interpretation?
Baglin et al. 2010 [11]	H	Y	Y	Y	Y	Y
Bassi. 2011 [30]	H	Y	Y	Y	Y	Y
Peters et al. 2015 [31]	H	Y	Y	Y	Y	Y
Stricklin. 2016 [32]	H	Y	Y	Y	Y	Y
Fereidouni et al. 2017 [33]	H	Y	Y	Y	Y	Y

*Scoring: Y=yes, N=no, C=can’t tell, H=high

## Results

**Fig. 1:**
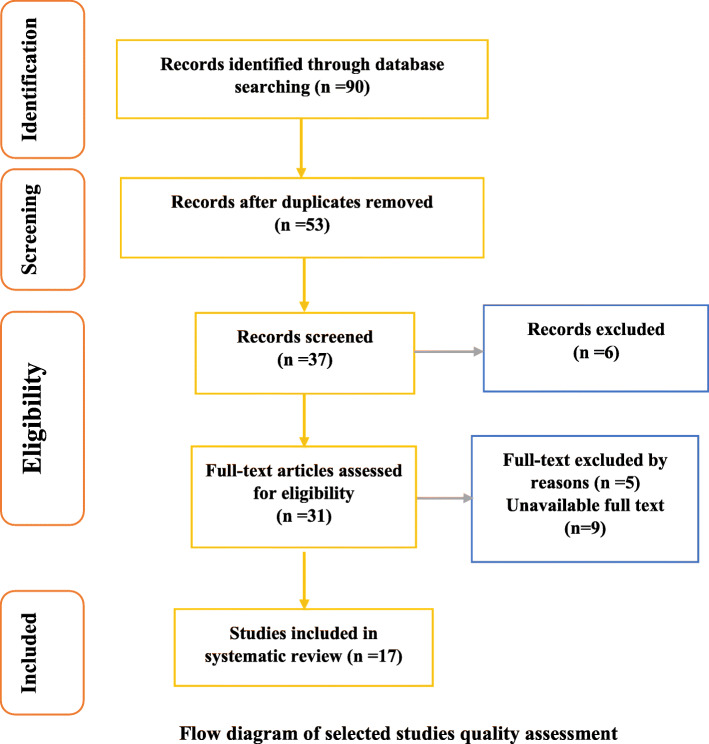
Flow diagram of selected studies quality assessment

This systematic review was reported based on the PRISMA guidelines. The flowchart of the studies included in the review is shown in Fig. 1. Seventeen articles published during 2004-2020 were included: including five quasi-experimental studies, three descriptive studies, four mixed method studies, and five qualitative studies [11, 12, 19–33]. The total number of participants was 1,866, ranging from 14 to 613 in each study. The procedure for selecting studies using PRISMA diagrams is shown in Fig. 1.

In all quasi-experimental studies, the main intervention was community-based education. Community-based settings in these studies included homes, aid agencies, community sites, clinics, schools, child-care centers, nursing homes (homes for the Aged), addiction treatment centers, care centers for the people with disabilities, dental centers, screening centers, care-centers for the homeless and rural and suburban areas. Studies explores participants were from different countries (United States, Taiwan, Africa, Singapore, United Kingdom, Australia, Indonesia and Iran). In addition, one of the combined studies included a community-based education intervention in a small portion of the study. In this type of study, students’ experiences of community-based education and their skills are examined. Qualitative studies also examined students’ experiences of community-based education.

### Effect of community-based education

The quasi-experimental studies in this systematic review had a pretest and a posttest, but only the Nowak study had a control group and the other studies were single-group studies [19]. Their results were statistically significant (*p *<0.05) [11, 19, 20, 22, 24, 27, 31, 32]. In all studies, community-based education affected the skills of undergraduate nursing students. The main findings (Table 3) show that community-based education of undergraduate nursing students enhanced professional skills in eight studies. Six undergraduate nursing students participated in the Baglin study and experienced a variety of community-based practice placements. The results of interviews with students led to four topics. These include to students’ basic skills acquisition and practice, the development of their working relationships with educators, patients and others, the learning opportunities offered by practice placement and the effect of such a placements on their confidence to practice [11]. The Nowak study was a quasi- experimental study using one way RMANOVA. Group mean outcomes measures were compared in three time periods, before to the programs, immediately after the program and in two weeks. Normality of distribution, homogeneity of variance, and random allocation of both groups was established. The disaster preparedness skills scores were measured in both groups. The results of survey showed that a statistically significant skill improvement between the treatment group and control groups (*p *<0.05) [19]. In the Lubber’s study, students assessed their confidence on 16 items. Paired t-tests were performed to compared students’ confidence in their pediatric knowledge and skills as assessed at pre-test and at post-test. When evaluating the full 16-item scale, students’ confidence increased significantly from pre-test (M =2.39, SD =0.65) to post-test (M = 4.13, SD = 0.37), (*p *<0.01). Each of the four item sub-scales, knowledge, skills communication, and documentation showed significant increases in students’ confidence from pre-test to post-test. Four additional items of the perceived confidence in pediatric nursing knowledge and skills questionnaire addressed student satisfaction with learning. Student reported a high level of satisfaction (M = 4.36, SD = 0.50) with their simulation experience [20]. In the Higgins study, students’ oral health knowledge and skills improved after completing the learning unit. The average pretest knowledge score was 66 % and the average posttest score was 86 %. In addition, their perceptions of the importance of building collaborative relationships with dental health providers increased. 99 % of the students strongly agreed that the educational unit was an effective way to learn oral health content. 97 % felt better prepared for interprofessional practice. They described the learning opportunity as useful and stated that their nursing practice would change as a result of their new knowledge [22]. In the De Villiers study, 61 % of participants reported positive experiences with community-based education and indicating that the program was effective in improving their skills [24]. In the Mwanika study, the qualitative results from the focus group discussions are presented under major themes namely: management and coordination of community-based education and service educational program, community-based education and service contribution to development of confidence and competence as health workers, professionalism and teamwork, willingness to work in rural health facilities and practice of primary health care. In addition, the quantitative findings in the Mwanika study showed that community-based education and service impact on the student with respect to development of confidence, professionalism, sense of responsibility, willingness to work in rural areas and primary health care skills [27]. Findings from the Peters study interview were presented on four topics: autonomy in practice, working with highly skilled nurses, focusing on holistic care and showing genuine interest in educating students [31]. The Stricklin study found that student nurses perceived that they were able to achieve learning outcomes and competency in the maost of psychiatric mental health nursing skills through experiences provided in community-based clinical settings. Three themes emerged from the data: meeting the challenges of developing psychiatric mental health nursing skills, sharing multiple experiences of competency, and empowering all nurses through psychiatric mental health nursing skills [32].

**Table 3:** Overview of all included studies in systematic review

Author, year	Location	Study type	Participants	Sample size(n)	Community-based education approach	Measure	Data collection	Main outcome
Nowak. 2015 [19]	United States	quasi-experimental Pre-test and post-test Two groups (control group)	baccalaureate nursing students	58	A community-based pilot program for disaster preparedness and partnerships with relief agencies	Questionnaire (Data were collected at 3 times before, after and 2 weeks.)	experimental and comparison groups at three time periods	Community-based education effectively promotes main disaster preparedness skills such as communicating with families, participating in volunteer activities, and professional skills for students. P<0.05
Lubbers et al. 2016 [20]	United States	quasi-experimental Pre-test and post-test Single group	baccalaureate nursing students	54	Community-based simulated experiences training course	Questionnaire	Perceived Confidence in Pediatric Knowledge and Skills Questionnaire at both pre-test and post-test intervals	Community-based curriculum increases nursing students ‘satisfaction with the curriculum and increases students’ confidence in knowledge, skills, and communication. P<0.001
Lubbers et al. 2017 [21]	United States	quasi-experimental Pre-test and post-test Single group	baccalaureate nursing students	61	Community-based simulated experiences training course	Questionnaire	Student Satisfaction and Self-Confidence Scale, Educational Practice Scale for Simulation, Simulation Design Scale	Community-based curriculum increases students’ confidence, satisfaction, knowledge and deep awareness. P<0.05
Higgins.2020 [22]	United States	quasi-experimental Pre-test and post-test Single group	baccalaureate nursing students	69	An online educational program about oral health	Questionnaire	pre and post assessment questionnaires to measure knowledge, skills, and attitudes	Increase skills and knowledge Improving the attitude of nursing students towards oral health
Cheng et al. 2020 [23]	Taiwan	quasi-experimental Pre-test and post-test Single group	baccalaureate nursing students	103	An experiential learning program includes activities related to infants, pregnant women, and the elderly in the community	Questionnaire	The pre-test data were collected at the semester start date, and the post-test data were collected at the end of the semester (eighteen weeks later)	Strengthen self-reflection Increase critical thinking Boost confidence P<0.01
De Villiers et al. 2004 [24]	South Africa	Descriptive	baccalaureate nursing students, Facilitators	53 Students 5 Facilitators	Students have received community-based and problem-based education programs in the past	Questionnaire	unstructured feedback, Interviews using the semi-structured questionnaire	Increasing the self confidence Improving professional and clinical skills Increasing teamwork
Ibrahim.2010 [12]	United States	Descriptive	Education (21 %), Sociology/Social Work (19 %), Psychology (9 %), Business/Health Care Administration (6 %), Biology/Pre-Med (6 %), Political Science (3 %), Communication (3 %), and Nursing (3 %)	176	Students have received community-based and problem-based education programs in the past	Questionnaire	Students filled out the survey in the classroom during the last week of Fall 2008 classes	Improving personal and communication skills Increase critical thinking
Wee et al. 2010 [25]	Singapore	Cross-sectional	baccalaureate nursing students, Medical students	37 Nursing students 576 Medical students	Students have received a community-based learning project in the past	Questionnaire	using a self-administered anonymised questionnaire in Public Health Screening	Community-based learning projects improve communication skills, teamwork, leadership skills, critical thinking skills, and practical skills, the ability to identify community social issues, and the ability to use knowledge and understand challenges. p<0.05
Street et al. 2007 [26]	England	Mixed method	baccalaureate nursing students, Medical students	66 Nursing students 94 Medical students	A two-week program to visit a child with a disability at home and at school. There is a control group.	Questionnaire	Feedback, focus group	Community-based professional learning experiences enhance students’ self-confidence and communication skills and a positive attitude, especially among nursing students.
Mwanika et al. 2011 [27]	Africa	Mixed method	Nursing students, pharmacists and doctors and dentists students	150	Participants have already received a community-based service and training program	Questionnaire	focus group (Manifest content analysis)	A community-based educational and service program has a positive impact on students and promotes their self-confidence and skills in careers, management, teamwork, communication skills, and care competencies.
Parry et al. 2018 [28]	Australia	Mixed method	baccalaureate nursing students, staff and clinical supervisor	T:14 8 Students 6 Staff	Students took an educational program in community settings.	Questionnaire	interview	Social spaces greatly increase nursing students’ understanding of the impact of health conditions on people. Also, community-based education and being in the community increase understanding, experience, extensive knowledge, expertise and increase decision-making skills and self-confidence in nursing students.
Lestari et al. 2020 [29]	Indonesia	Mixed method	Nursing, midwifery and medical students	84 Nursing students 61 Midwifery 109 Medical students	Students received a community-based inter-professional training program.	Questionnaire	focus group	Improving teamwork skills and practical skills Improving students’ communication skills with the community
Baglin et al. 2010 [11]	United Kingdom	Qualitative	baccalaureate nursing students	6	Students received a community-based training program for 12 weeks.	descriptions of participants’ experiences	semi-structured, contemporaneous, reflective diaries	Nursing students’ learning experiences on community-based sites enhance basic skills, develop relationships with instructors, patients, and others in the community, as well as boost their self-confidence.
Bassi. 2011 [30]	United States	Qualitative	undergraduate nursing students	18	Students took part in a pilot service-learning project targeting tobacco use in a local elementary school.	students were assessed on the basis of their teaching plans and structured reflection assignments	structured interviews, survey, sets of reflective assignments	Community-based learning program promotes academic, social and personal development, competence, independence, teamwork skills and community participation (interpersonal relationships) in students.
Peters et al. 2015 [31]	Australia	Qualitative	pre-registration nursing students	9	Students are trained in community-based clinical settings	interviews were conducted via telephone	Semi structured interviews, audio-recorded	Community-based educational sites and students’ experiences in these areas lead to performance independence, skills development and interest in them.
Stricklin. 2016 [32]	United States	Qualitative	baccalaureate nursing students, faculty members	42 Students4 faculty members	Students received a community-based inter-professional training program.	questionnaires	Semi-structured interviews, focus groups, and campus and clinical site visits	Improving clinical competence and skills increasing the self confidence
Fereidouni et al. 2017 [33]	Iran	Qualitative	Nursing students and professors	17 Students3 Professors	The community based training and internship planning conducted for one year	This study was conducted using the content analysis approach	Semi-structured interviews, field notes, five focus groups	Improving communication Promoting professional competence

In nine studies, community-based education improved communication skills with educators, patients, community members, children, adolescents, the elderly, people with disabilities, family, patient relatives, and other health professionals [11, 12, 19, 20, 25–27, 29, 33]. Nine studies mentioned increasing self-confidence [11, 20, 21, 23, 24, 26–28, 32] and five studies mentioned increasing knowledge and awareness [20–22, 25, 28]. Promoting teamwork skills was mentioned in four studies [25, 27, 29, 30] and improving thinking skills through education was mentioned in three studies [11, 23, 25]. The content of the community-based curriculum and the strategy for its implementation varied across studies, but all studies were conducted in community-based settings. Educational programs included community-based learning projects, community-based simulated experiences, community-based pilot programs, and courses in clinical settings.

## Discussion

The purpose of this systematic review was to examine the impact of community-based education on the undergraduate nursing students’ skills. After reviewing 17 selected articles from the United States (7 articles), United Kingdom (2 articles), Australia (2 articles), Africa (2 articles), Taiwan (1 article), Singapore (1 article), Indonesia (1 article), and Iran (1 article), the findings were summarized in relation to the impact of community-based education on nurses’ skills. Community-based education can be said to be as one of the most effective educational methods for improving the skills of undergraduate nursing students. The results of the present study were compared with those of other studies.

The findings of this systematic review indicate that community-based education promotes the development of professional skills in nursing students [11, 19, 20, 22, 24, 27, 31, 32]. Research has shown that the use of community experiences in educational programs enhances professional skills [10]. Community-based education develops occupational competencies and skills such as problem-solving, leadership, and management [9]. In addition, community learning experiences promote competencies needed by students [15]. The findings of the present study are consistent with other studies [11, 24, 34, 35].

Students in a community-based curriculum are exposed to a variety of challenging situations, such as home visits, school visits, visiting and caring for people with disabilities, and interacting with diverse people. Therefore, they develop their skills in dealing with diverse populations [36]. Students acquire the ability to identify health problems in the community, work with available resources in the community, and provide care that is appropriate to the context and culture of the community [34].

The results of this systematic review also indicate that community-based education was rated as useful by faculty, students, and clients. Providing community health services to work with students in the real context of society and among people increases their personal skills and abilities, including improving their communication skills with professors, instructors and the community [11, 12, 19, 20, 25, 27, 33]. The results of many studies are consistent with the findings of the present study [2, 9, 11, 15, 24, 37–39]. Community-based education strengthens students’ communication skills when interacting with professionals and the community with clients and professionals. Communication skills and interpersonal relationships are important skills that are considered essential in order to practice an effective and efficient profession in society. In this program, students progressively develop their communication skills [9]. Communication and negotiation skills are necessary to build relationships in the community, to work effectively with the physician and other members of the medical team, and to educate patients. This high level of communication skills is the focus of these programs [40].

The results of the present systematic review suggest the use of community-based teaching enhances the confidence of undergraduate nursing students. Researchers found that engaging nursing students in the community and confronting their problems increased students’ confidence in caring for people in the community [11, 20, 21, 23, 24, 26–28, 32]. The findings of the present study are consistent with the findings of other studies. For example, in a study in South Africa and Uganda, a large percentage of nursing students reported that practicing skills in real-life communities increased their confidence [9, 24]. These findings have also been confirmed in other studies [11, 41–43], for example, that increasing the level of skills, awareness, and community involvement, working in interdisciplinary teams, and being self-reliant in this educational program increases students’ self-confidence [9].

The results of this systematic review have shown that community-based education is an effective way to raise awareness and provide necessary experiences for nursing students [20–22, 25, 28]. Being in the community greatly increases nursing students’ knowledge and understanding of the impact of health conditions on the population. As nursing students provide care to vulnerable groups, they gain many experiences interacting with diverse populations, which deepen their knowledge and awareness. Findings from other studies support the findings of the present study [9, 15, 24, 39, 40]. A community-based curriculum exposes nursing students to the impact of living conditions and other realities where students can relate theory to the real world. This makes learning more meaningful and strengthens their experiences and knowledge. Students become more aware of social problems and inequalities in health care and other factors that affect health [34].

Similar to other studies, the results of the present systematic review showed that community-based education improved teamwork skills in nursing students [24, 25, 27, 29, 30]. Nursing students considered this training program to be successful, and felt that it enhanced their group activities and teamwork skills. The results of other studies were consistent with the findings of the present study [15, 38]. Nursing students often have limited opportunities on campus or in the clinic to participate in teamwork. The scope of community-based sites may provide students with opportunities to learn group work and inter-professional work so that students learn how to work effectively and efficiently in a professional team [35].

This systematic review shows that community-based education creates a real and interactive learning environment, and that students develop critical thinking skills during instructor-led activities [12, 23, 25]. community-based education has been shown to promote critical thinking in nursing students because of its characteristics, such as the emphasis on the learner exploration of problems and the use of evidence in problem solving [9, 44–46]. In this way, students interpret their diverse experiences based on what they encounter, hear, read and see [9].

Community-based education provides an opportunity for students to apply their theory and knowledge in a real and practical environment. Community-based education increases their self-confidence and satisfaction [20] and all students with different nursing roles ( Clinic nurse, school nurse, home care nurse, district nurse) get acquainted and gain different experiences, while those who are in one place are able to gain less experience [21]. As a result, students understand the importance of the program and make changes in their performance [22]. During the community-based training program, students receive feedback and reflection as well as work with different teams. This allows them to reflect and develop their critical thinking skills, communication skills and teamwork skills [23].

The studies presented had limitations. The quasi-experimental studies used convenience samples, and only one of them had a control group, and it is not certain whether the difference between pre-test and post-test was solely due to the training course. However, the validity and reliability of the questionnaires used in the studies were found to be high [19–23].

## Limitations

In this systematic review there were restrictions on access to the original articles due to the sanctions in Iran, for example, access to the full text of 9 articles was not possible. Considering the findings and the positive impact of community-based education on undergraduate education, it is suggested that community-based education in clinical education in hospitals and clinics should also be reviewed.

## Conclusions

In community-based education, students are confronted with the real life problems in the context of society. This enables them to deal with problems and gradually develops vocational skills, communication skills, critical thinking and teamwork skills. In addition, this type of education strengthens the learning process of students and leads them to gain experience and sound knowledge about health issues in the community, while increasing their self-confidence. According to the findings of the studies reviewed in this review on the effectiveness of community-based education on the undergraduate nursing students’ skills, community-based education can be used as an effective pedagogical approach in curriculum and program development. Since community-based education is an approach that has recently received special attention, it is clearly necessary to conduct community-based studies with appropriate methodology and stronger evidence to confirm the findings of the present study.

## Data Availability

All data generated or analyzed during this study are included in this published article.

## Abbreviations

MMAT: Mixed Methods Appraisal Tool
PRISMA: Preferred Reporting Items for Systematic Reviews and Meta-Analyses
